# Status of institutional‐level respectful maternity care: Results from the national Ethiopia EmONC assessment

**DOI:** 10.1002/ijgo.13452

**Published:** 2020-12-31

**Authors:** Ephrem D. Sheferaw, Rena Bakker, Tefera Taddele, Abiyu Geta, Young‐Mi Kim, Thomas van den Akker, Jelle Stekelenburg

**Affiliations:** ^1^ Department of Health Sciences Global Health University Medical Center Groningen University of Groningen Groningen The Netherlands; ^2^ Ethiopian Public Health Institute Addis Ababa Ethiopia; ^3^ Ministry of Health Addis Ababa Ethiopia; ^4^ Jhpiego, an affiliate of Johns Hopkins University Baltimore MD USA; ^5^ Department of Obstetrics and Gynaecology Leiden University Medical Centre Leiden The Netherlands; ^6^ Athena Institute VU University Amsterdam The Netherlands; ^7^ Department of Obstetrics and Gynecology Leeuwarden Medical Center Leeuwarden The Netherlands

**Keywords:** Ethiopia, Mistreatment, Respectful maternity care

## Abstract

**Objective:**

To assess the availability of an institutional‐level respectful maternity care (RMC) index, its components, and associated factors.

**Methods:**

A cross‐sectional study design was applied to a 2016 census of 3804 health facilities in Ethiopia. The availability of an institutional‐level RMC index was computed as the availability of all nine items identified as important aspects of institutional‐level RMC during childbirth. Logistic regression analysis was used to identify factors associated with availability of the index.

**Results:**

Three components of the institutional‐level RMC index were identified: “RMC policy,” “RMC experience,” and “facility for provision of RMC.” Overall, 28% of facilities (hospitals, 29.9%; health centers, 27.8%) reported availability of the institutional‐level RMC index. Facility location urbanization (urban region), percentage of maternal and newborn health workers trained in basic emergency obstetric and newborn care, and availability of maternity waiting homes in health facilities were positively associated with availability of the institutional‐level RMC index.

**Conclusion:**

Only one in three facilities reported availability of the institutional‐level RMC index. The Ethiopian government should consider strengthening support mechanisms in different administrative regions (urban, pastoralist, and agrarian), implementing the provision training for health workers that incorporates RMC components, and increasing the availability of maternity waiting homes.

## Synopsis

In Ethiopia, only one in three facilities reported availability of an institutional‐level respectful maternity care index. Factors associated with availability of the index were identified.

## 1 INTRODUCTION

Mistreatment of women in health facilities during labor and childbirth has been recognized as a global problem.^1^, ^2^ The causes of mistreatment during childbirth are complex, embedded within a sociocultural context and shaped by characteristics of health facilities and care providers.^3^, ^4^ The WHO categorizes mistreatment of women into seven domains: (a) physical abuse, (b) sexual abuse, (c) verbal abuse, (d) stigma and discrimination, (e) failure to meet professional standards of care, (f) poor rapport between women and providers, and (g) health system conditions and constraints.^1^

Several research groups, using various measurement criteria, found that 21%–79% of women experience mistreatment during childbirth in Ethiopia.^5^ None of the studies conducted in Ethiopia focused on health system conditions as a component of mistreatment; however, the role of institutional characteristics deserves special attention because it affects a healthcare provider's behavior and attitude to providing respectful care.^6^ For example, an unfavorable health facility environment is likely to increase stress levels among healthcare providers, resulting in mistreatment of women during childbirth.^7^

In 2016, the Ethiopian government launched its Health Sector Transformation Plan, which aims to improve maternal and newborn health outcomes by promoting compassionate and respectful care.^8^ A key strategy to achieve this goal comprises health resource facilitation, such as the rollout of maternity waiting homes (MWHs), which provide accommodation for pregnant women in close proximity to the health facility.^8^, ^9^, ^10^ MWHs are usually constructed with community participation and managed by the health facility. Another important approach encompasses the provision of countrywide emergency obstetric and newborn care (EmONC), which includes life‐saving interventions for the main causes of maternal and neonatal morbidity and mortality.^11^

Understanding contributors to institutional‐level respectful maternity care (RMC) during childbirth will help to maximize the effectiveness of RMC interventions. It may also positively influence the utilization of maternity services. To our knowledge, potential components of institutional‐level RMC have not been systematically assessed before. Therefore, the primary aim of the present study was to describe an institutional‐level RMC index. Secondary aims were to (a) identify components of the institutional‐level RMC index during childbirth, (b) assess levels of the institutional‐level RMC index and components in hospitals and health centers in Ethiopia; and (c) determine institutional‐level factors associated with the reported institutional‐level RMC prerequisites.

## 2 MATERIALS AND METHODS

### 2.1 Study design

The present study used a subset of the 2016 EmONC assessment data that focus on health facility level policies, norms, and practices that affect provision of RMC. The EmONC assessment utilized a cross‐sectional census of all health facilities in Ethiopia that provided childbirth services prior to the assessment.^12^, ^13^ The study protocol was reviewed and approved by the Scientific and Ethical Review board of the Ethiopian Public Health Institute. Each study participant gave informed oral consent prior to participation.

### 2.2 Study setting

The study included all private and public health facilities (hospitals, health centers, maternal and child health [MCH] specialty centers, MCH specialty clinics, and higher clinics) across all nine regions and two city administrations in Ethiopia. All health facilities that had a mandate to provide childbirth services according to national accreditation agency criteria confirmed that births had taken place in the 12 months preceding the assessment and were functional during the data collection period.

### 2.3 Data collection

The data collection tools were adapted to the Ethiopian context from the 2008 EmONC assessment tool and the 2014 Averting Maternal Death and Disability tools. The analyzed data were extracted from modules one and two (“facility identification” and “infrastructure and human resources”).^14^ Tool adaptation took place in a workshop attended by local experts who ensured that the Ethiopian context was considered in the questionnaire. Three rounds of pilot testing were conducted to ensure a proper flow of questions, estimate the length of time required for interviews, and identify issues related to the understanding of terms and concepts in the electronic data entry program. Identified inconsistencies were corrected.

The data collectors had at least a bachelor's degree in a health‐related field. All data collectors attended 10 days of training and worked in teams of three, with one group member serving as team leader. Field level data collection was conducted from approximately May 15 to December 15, 2016. Data were collected by interviewing health facility and maternity unit heads in a private area at the facility. The data collectors also observed the availability of facilities necessary for provision of RMC, such as curtains, waiting areas, and bathrooms.

### 2.4 Data quality

To ensure accurate data quality, pre‐ and post‐tests were administered to data collectors to assess their learning and understanding of assessment guidelines and standards for data collection. Team leaders reviewed all completed questionnaires to ensure completeness. Regional and national coordinators visited and communicated with data collection teams to provide support and help when difficulties arose at individual facilities. Data were analyzed using Stata version 14 (Stata Corp., College Station, TX, USA).

### 2.5 Variables and data analysis

The “institutional‐level RMC index” was defined as the health facility's availability of physical infrastructure, equipment, policies, and norms that together enable women to experience RMC during childbirth services. It includes a physical infrastructure that encourages privacy and confidentiality, availability of waiting area for companions, availability of bathrooms, and facility‐related policies and norms to ensure a positive experience during labor and childbirth.

In the first step, 11 items (questions) measured in a binary (yes/no) format were identified that highlight important aspects of institutional‐level RMC during childbirth: “women can choose a companion of their choice,” “women can choose birthing position,” “women can walk around during labor,” “availability of curtains for privacy,” “availability of waiting areas for women and companions,” “availability of functioning toilets for companions,” “availability of food for women,” “women have never shared beds before or after birth,” “women have never slept on the floor,” and “women have never given birth on the floor.”

**FIGURE 1:**
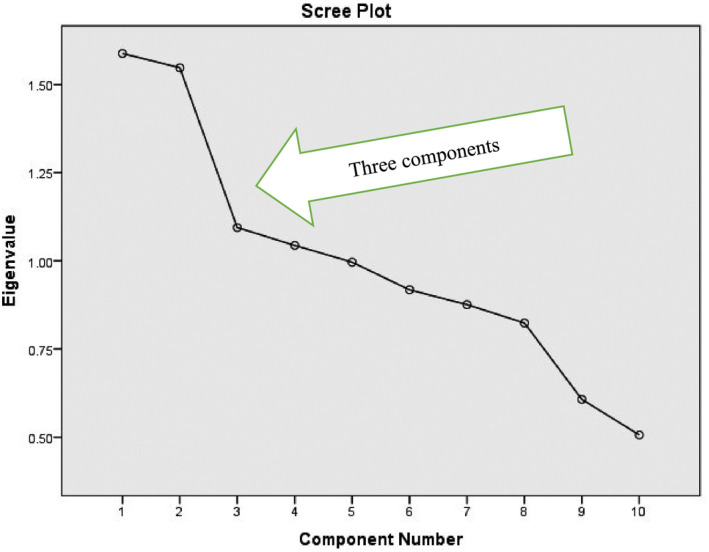
Scree plot used to determine number of items to retain in the principal component analysis

Two items (“woman can walk around during labor” and “availability of food for women”) were excluded from the principal component analysis due to low factor loadings (<0.35), although one item was retained owing to technical relevance even though it did not fulfill statistical criteria. The nine remaining items that measured specific aspects of institutional RMC during childbirth were grouped into components (Table 1). Three components were extracted by using scree plot criteria,^15^ which are used to identify the number of factors to retain in a principal component analysis (see File S1 for communalities, total variance explained, and rotated component matrix). These components were labeled “policy,” “facility,” and “experience” (Figure 1).

**TABLE 1:** Component matrix for the principal component analysis^a^

**RMC item**	**Component**		
	**1**	**2**	**3**
Allow companion during childbirth	0.794		
Allow women to have a female companion during labor	0.729		
Allow women to choose their preferred position during labor/childbirth	0.595		
Women have never slept on the floor		0.784	
Women have never given birth on the floor		0.7	
Women have never shared beds before or after childbirth		0.625	
Waiting area for companion's use			0.733
Functioning toilet for companion's use			0.715
Curtains/means of providing privacy			0.335

Abbreviation: RMC, respectful maternity care.

^a^ ^^Three components extracted.

The component “policy” was calculated from three items (“women can choose a companion of their choice,” women can choose a birthing position,” and “women can walk around during labor”) and labeled as available when all three items were reported as yes. The component “facility” was calculated from three items (“availability of curtains for privacy,” “availability of waiting areas for women and companions,” and “availability of functioning toilets for visitors and family use”) and considered as available when all three conditions were observed or reported as yes. The component “experience” was calculated from three items (“women have never shared beds before or after birth,” “women have never slept on the floor,” and “women have never given birth on the floor”) and considered to be available when all three items were reported as yes by the maternity unit lead. The variable institutional‐level RMC index was calculated as a composite score of all nine items. The institutional‐level RMC index was considered to be available only if all nine items were available.

In the second step, multivariate logistic regression was used to identify factors associated with the availability of the institutional‐level RMC index during childbirth, which was the outcome variable. Covariates identified from other studies included managing authority, administrative region type, ratio of births to maternity beds, ratio of childbirth to maternity healthcare workers, proportion of MCH providers trained in BEmONC, and availability of MWHs in health facilities.^3^, ^7^

The variable “ratio of births per year to maternity beds” indicated the level of crowding at the facility, whereas”the ratio of childbirth to maternity healthcare workers” was applied as a measure of the workload of providers. Continuous explanatory variables (number of childbirths to maternity beds, number of childbirths to maternity unit assigned health workers and proportion of MCH providers trained in BEmONC) were categorized into four quartiles to facilitate data interpretation. Frequencies were calculated for a range of facility factors. The data are presented as odds ratio (OR) or adjusted odds ratio (aOR), combined with 99% confidence intervals (CIs) and *P* values to compensate for multiple testing.

## 3 RESULTS

Among 4385 private and public health facilities in Ethiopia, 11 were excluded from the assessment due to civil unrest in their catchment areas, 568 were excluded due to absence of service during the 12 months preceding the survey and 2 refused to participate in the assessment. Overall, 3804 facilities (336 health centers and 3488 hospitals) were included in the analysis.

The background characteristics of the health facilities are summarized in Table 2. Most hospitals (n = 236, 74.7%) were public institutions, some (n = 61, 19.3%) were private for‐profit institutions, and a few (n = 19, 6.0%) were private not‐for‐profit institutions. Nearly all health centers (n = 3426, n = 98.2%) were public institutions. Most hospitals (n = 235, 74.4%) and health centers (*n* = 3074, 88.1%) were found in agrarian regions. Most hospitals (n = 293, 92.7%) were in urban areas, whereas most health centers (n = 2284, 65.5%) were in rural areas. The majority of hospitals had the lowest delivery volume (≤27 births per maternity bed annually) and were the least crowded (≤31 births per MNH provider).

**TABLE 2:** Background characteristics of health facilities disaggregated by facility type^a^

**Facility type**	**Hospitals (n = 316)**	**Health centers (n = 3488)**	**Total (n = 3804)**
Managing authority			
Public	236 (74.7)	3426 (98.2)	3662 (96.3)
Private, for‐profit	61 (19.3)	22 (0.6)	83 (2.2)
Private, not‐for‐profit	19 (6)	40 (1.1)	59 (1.6)
Region^b^			
Agrarian	235 (74.4)	3074 (88.1)	3309 (87)
Pastoralist	21 (6.6)	287 (8.2)	308 (8.1)
Urban	60 (19)	127 (3.6)	187 (4.9)
Facility location			
Urban	293 (92.7)	1204 (34.5	1497 (39.4
Rural	23 (7.3)	2284 (65.5	2307 (60.6
Annual births			
<52	47 (14.9)	244 (7.0)	291 (7.6)
52–182	38 (12.0)	557 (16.0)	595 (15.6)
183–365	22 (7.0)	899 (25.8)	921 (24.2)
366–499	22 (7.0)	536 (15.4)	558 (14.7)
500–999	63 (19.9)	1013 (29.0)	1076 (28.3)
≥1000	124 (39.2)	239 (6.9)	363 (9.5)
Births per maternity bed			
1st quartile (≤27)	136 (43.2)	810 (23.3)	946 (25.0)
2nd quartile (28–50)	85 (27.0)	863 (24.9)	948 (25.1)
3rd quartile (51–85)	74 (23.5)	871 (25.1)	945 (25.0)
4th quartile (≥86)	20 (6.3)	925 (26.7)	945 (25.0)
Births per MNH provider			
1st quartile (≤31)	124 (40.1)	811 (23.7)	935 (25.0)
2nd quartile (32–63)	80 (25.9)	852 (24.9)	932 (25.0)
3rd quartile (64–117)	72 (23.3)	861 (25.1)	933 (25.0)
4th quartile (≥118)	33 (10.7)	900 (26.3)	933 (25.0)
MNH providers trained in BEmONC			
1st quartile (≤14)	118 (38.2)	912 (26.6)	1030 (27.6)
2nd quartile (14–28)	51 (16.5)	864 (25.2)	915 (24.5)
3rd quartile (29–49)	57 (18.4)	931 (27.2)	988 (26.5)
4th quartile (≥50)	83 (26.9)	717 (20.9)	800 (21.4)
Maternity waiting home or room			
No	258 (81.6)	1545 (44.3)	1803 (47.4)
Yes, room in facility	39 (12.3)	1196 (34.3)	1235 (32.5)
Yes, freestanding	19 (6.0)	747 (21.4)	766 (20.1)

Abbreviations: BEmONC, basic emergency obstetric and newborn care; MNH, maternity and newborn health.

^a^ Values are given as number (percentage).

^b^ Agrarian regions: Tigray, Amhara, Oromia, and SNNP. Pastoralist regions: Afar, Somali, Benishangul‐Gumuz, and Gambela. Urban regions: Addis Ababa, Harari, and Diredawa.

Table 3 summarizes the institutional‐level RMC index of the health facilities. Overall, 29.9% (n = 94) of hospitals and 27.8% (*n* = 969) of health centers fulfilled the institutional‐level RMC index. As compared with hospitals, health centers reported higher rates of RMC‐related policies (54.3% [*n* =1892] vs 49.4% [n = 156], *P* = 0.092) and facility‐level RMC experience (83.1% [n =2897] vs 75.6% [n =239]; *P* = 0.001). In terms of availability of the facility component necessary for the provision of RMC, hospitals reported better performance than health centers (76.5% [n =241] vs 58.9% [n =1998]; *P* < 0.001).

**TABLE 3:** Prevalence of institutional‐level RMC index components^a^

**Component**	**Hospital/MCH specialty center (n = 316)**	**Health center/clinic (n = 3488)**	**Total (n = 3,804)**	***P* value**
RMC index				
No	220 (70.1)	2512 (72.2)	2732 (72.0)	0.428
Yes	94 (29.9)	969 (27.8)	1063 (28.0)	
Policy				
No	160 (50.6)	1592 (45.7)	1752 (46.1)	0.092
Yes	156 (49.4)	1892 (54.3)	2048 (53.9)	
Experience				
No	77 (24.4)	589 (16.9)	666 (17.5)	0.001
Yes	239 (75.6)	2897 (83.1)	3136 (82.5)	
Facility				
No	74 (23.5)	1397 (41.1)	1471 (39.6)	<0.001
Yes	241 (76.5)	1998 (58.9)	2239 (60.4)	

Abbreviation: RMC, respectful maternity care.

^a^ ^^Values are given as number (percentage).

After adjusting for the effects of managing authority, urban rural status, proportion of MCH providers trained in BEmONC, the ratios of number of childbirth per available beds and providers, and availability of MWHs in health facilities, the likelihood of health facilities fulfilling the institutional‐level RMC index was higher for those located in urban regions than for those located in agrarian regions (aOR, 1.46; 99% CI, 0.91–2.34; *P* = 0.037), although the difference was not statistically significant (Table 4) .

**TABLE 4:** Association of facility characteristics with institutional‐level RMC index.

**Characteristic**	**Univariate analysis**		**Multivariate analysis**	
	OR (99% CI)	*P* value	aOR (99% CI)	*P* value
Facility type				
Hospital	Ref.			
Health center	0.90 (0.65–1.26)	0.428	0.89 (0.6–1.32)	0.448
Managing authority				
Public	Ref.			
Private, for‐profit	1.42 (0.78–2.59)	0.135	1.32 (0.65–2.7)	0.319
Private, not‐for‐profit	1.05 (0.50–2.21)	0.868	1.21 (0.56–2.64)	0.519
Region				
Agrarian	Ref.			
Pastoralist	0.79 (0.55–1.14)	0.099	0.97 (0.63–1.47)	0.835
Urban	1.49 (0.99–2.23)	0.012	1.46 (0.91–2.34)	0.037
Facility location	fi			
Urban	Ref.			
Rural	0.85 (0.7–1.03)	0.028	0.96 (0.77–1.18)	0.597
Births per MNH provider				
1st quartile (≤31)	Ref.			
2nd quartile (32–63)	1.01 (0.77–1.32)	0.947	0.94 (0.68–1.28)	0.588
3rd quartile (64–117	1.31 (1.01–1.71)	0.008	1.15 (0.82–1.62)	0.285
4th quartile (≥118)	1.14 (0.87–1.49)	0.199	0.95 (0.65–1.40)	0.748
Births per MNH bed				
1st quartile (≤27)	Ref.			
2nd quartile (28–50)	1.03 (0.79–1.35)	0.770	0.94 (0.68–1.28)	0.590
3rd quartile (51–85)	1.04 (0.79–1.35)	0.738	0.96 (0.68–1.36)	0.775
4th quartile (≥86)	1.19 (0.91–1.54)	0.094	1.13 (0.78–1.63)	0.398
MNH providers trained in BEmONC				
1st quartile (<14%)	Ref.			
2nd quartile (14–28%)	1.83 (1.39–2.40)	<0.001	1.75 (1.32–2.31)	<0.001
3rd quartile (29–49%)	1.78 (1.36–2.33)	<0.001	1.74 (1.32–2.29)	<0.001
4th quartile (≥50%)	1.89 (1.43–2.50)	<0.001	1.84 (1.36–2.48)	<0.001
Maternity waiting home or room				
No	Ref.			
Yes, room in facility	1.13 (0.91–1.40)	0.144	1.14 (0.90–1.44)	0.157
Yes, freestanding	1.35 (1.06–1.72)	0.001	1.41 (1.08–1.84)	0.001

Abbreviations: aOR, adjusted odds ratio; BEmONC, basic emergency obstetric and newborn care; CI, confidence interval; MNH, maternity and newborn health; OR, odds ratio; RMC, respectful maternity care.

Facilities in the higher (second, third, and fourth) quartiles of providers trained in BEmONC were more likely to fulfill the institutional‐level RMC index: aOR, 1.75 (99% CI, 1.32–2.31; *P* < 0.001), 1.74 (99% CI, 1.32–2.29; *P* < 0.001), and 1.84 (99% CI, 1.36–2.48; *P* < 0.001), respectively. Lastly, facilities with freestanding MWHs were 41% more likely to fulfill the institutional‐level RMC index as compared with those with no MWHs (aOR, 1.41; 99% CI, 1.08–1.84; *P* = 0.001).

## 4 DISCUSSION

In the present study, facilities in urban regions, facilities with a higher proportion of MNH providers trained in BEmONC (quartiles 2–4), and facilities with freestanding MWHs were significantly associated with higher availability of the institutional‐level RMC index. In line with previous research, the institutional‐level RMC index was found to comprise three components: policy, facilities, and experience. The components policy and facilities were also included as health systems conditions and constraints in a 2015 systematic review of mistreatment during childbirth.^16^

The finding that facilities in urban regions performed better on availability of the institutional‐level RMC index might be due to the better provision of resources in urban areas.^17^ Alternatively, the availability of health administrative structure in urban areas might help facilities to get closer supervision and support. A study on service availability and readiness conducted in 2014 in Ethiopia showed that only 6%–14% of health facilities in agrarian regions received supervision in the 6 months preceding the survey, as compared with 24%–50% in urban regions.^17^

The finding that facilities with a higher proportion of MNH providers trained in BEmONC (quartiles 2–4) had higher availability of the institutional‐level RMC index might be attributed to both the fact that RMC is included in the national BEmONC training package,^18^ and the continued effort of the Ethiopian Ministry of Health (MOH) in implementation of the compassionate, respectful, and caring (CRC) agenda.^19^ Since 2015, the Ethiopian MOH has integrated concepts of CRC into existing training packages including BEmONC, subsequent to having developed and implemented CRC in job training.^8^, ^19^ A previous study in Ethiopia has shown that facilities that implement a quality improvement program, focusing on training and mentoring on BEmONC, are associated with improved performance in labor‐ and childbirth‐related skills.^20^

The finding that facilities with freestanding MWHs were associated with availability of the institutional‐level RMC index might be related to strong community links and management of facilities. Construction of MWHs involves the involvement of both health facility management and the local community; thus, the provision of a MWH is an important indicator of a health facility's management commitment to improving maternity care access to women from distant areas. Establishing good community ties, managing health facilities better, and investing in maternity healthcare workers all show a higher awareness of women's needs and more willingness to improve care in order to attain RMC. The Ethiopian MOH standardized the implementation of MWHs in 2015 and supported its expansion across the country to improve access to childbirth service for women in rural areas.^21^ The finding is consistent with a study that analyzed the 2016 EmONC module on MWHs and reported a reduction in perinatal death and obstetric complication in facilities with MWHs as compared with those without.^12^ Similarly, a systematic review on the effect of MWH use in Ethiopia and other countries showed a reduction in maternal mortality and stillbirth rates.^22^

The present study has both strengths and limitations. An important strength is that it examined institutional‐level factors affecting the availability of RMC by assessing all facilities in the country. Its limitations encompass the fact that the number of items included to measure the overall RMC condition were selected from a small set of questions, as opposed to a longer list covering all theoretic aspects of institutional‐level RMC conditions. It should be acknowledged, however, that the limited items included to represent RMC were selected from published studies after conducting an expert review,^23^ which will allow replication of the study and might be considered as a strength. Another limitation involves the data collection method because health facility managers and maternity unit heads were interviewed on policy‐related questions: although the data collectors explained the purpose of the study, the responses might have been affected by desirability bias.

In conclusion, the present study found that the institutional‐level RMC index in health facilities comprised three components: policy, facilities, and experience. Urban administrative region, proportion of healthcare providers trained in EmONC, and availability of MWHs were associated with availability of the institutional‐level RMC index. Two in three health facilities did not have the institutional‐level RMC index in place. In line with its effort to provide a compassionate, respectful, and caring service, the study suggests that the Ethiopian government needs to consider strengthening support mechanisms in different administrative regions (i.e., urban, pastoralist, and agrarian), implement the provision of healthcare training that incorporates components of RMC, and increase the availability of MWHs. We recommend that the government should develop and implement RMC policies at the health facility level. The government also needs to support health facilities with the necessary resources to ensure availability of the necessary infrastructure and supplies for the provision of RMC.

## CONFLICTS OF INTEREST

The authors have no conflicts of interest.

## AUTHOR CONTRIBUTIONS

EDS contributed to study conceptualization, data analysis, original draft preparation, and manuscript editing. RB contributed to study conceptualization, original draft preparation, and manuscript editing. YMK, TVA, and JS contributed to the conceptualization, writing, review and editing of the manuscript. TT contributed to study design, and manuscript review and editing. AG contributed to manuscript review and editing. All authors read and approved the final manuscript.

## Supporting information

Table S1‐S2
