# Supplementary material for: Status of institutional‐level respectful maternity care: Results from the national Ethiopia EmONC assessment
Source: Int J Gynaecol Obstet. 2020 Dec 31;153(2):260–7. doi: 10.1002/ijgo.13452 (PMC8246788; doi:10.1002/ijgo.13452)
Supplement: Supplementary file 1 — Table S1‐S2 [file IJGO-153-260-s001.docx]

**TABLE S1** Communalities

|  | **Initial** | **Extraction** |
| --- | --- | --- |
| Allows woman to have a female companion during labor | 1 | 0.589 |
| Allows Companion during childbirth | 1 | 0.655 |
| Allows woman to walk around during labor | 1 | 0.14 |
| Allows women to choose her preferred position during labor/childbirth | 1 | 0.363 |
| Curtains/means of providing patient privacy | 1 | 0.157 |
| Functioning toilet for companions use | 1 | 0.582 |
| Food provided to patients by the facility | 1 | 0.025 |
| Women have never shared beds before or after childbirth | 1 | 0.393 |
| Women have never slept on the floor | 1 | 0.631 |
| Women have never given birth on the floor | 1 | 0.511 |
| Waiting area for companions use | 1 | 0.617 |

Extraction Method: Principal Component Analysis.

**TABLE S2** Rotated Component Matrix^a^

|  | | | |
| --- | --- | --- | --- |
| **RMC items** | **Component** | | |
|  | 1 | 2 | 3 |
| Allows Companion during childbirth | 0.838 |  |  |
| Allows woman to have a female companion during labor | 0.78 |  |  |
| Allows women to choose her preferred position during labor/childbirth | 0.584 |  |  |
| Women have never slept on the floor |  | 0.796 |  |
| Women have never given birth on the floor |  | 0.713 |  |
| Women have never shared beds before or after childbirth |  | 0.617 |  |
| Waiting area for companions use |  |  | 0.786 |
| Functioning toilet for companions use |  |  | 0.77 |
| Curtains/means of providing patient privacy |  |  | 0.391 |
| Extraction Method: Principal Component Analysis. | | | |
| Rotation Method: Varimax with Kaiser Normalization. | | | |
| a. Rotation converged in 4 iterations. | | | |
